# Supplementary material for: Modeling quarantine during epidemics and mass-testing using drones
Source: PLoS One. 2020 Jun 24;15(6):e0235307. doi: 10.1371/journal.pone.0235307 (PMC7314055; doi:10.1371/journal.pone.0235307)
Supplement: Attachment — Submitted filename: PLOS response - Google Docs.pdf [file pone.0235307.s001.pdf]

Journal Requirements:

When submitting your revision, we need you to address these additional requirements.

1. Please ensure that your manuscript meets PLOS ONE's style requirements, including those for file naming. The PLOS ONE style templates can be found at

https://journals.plos.org/plosone/s/file?id=wjVg/PLOSOne_formatting_sample_main_body.pdf and

https://journals.plos.org/plosone/s/file?id=ba62/PLOSOne_formatting_sample_title_authors_affiliations.pdf

---- We put the paper into PLOS latex template and followed PLOS formatting instructions.

2. We note that Figure 2 in your submission contain map images which may be copyrighted. All PLOS content is published under the Creative Commons Attribution License (CC BY 4.0), which means that the manuscript, images, and Supporting Information files will be freely available online, and any third party is permitted to access, download, copy, distribute, and use these materials in any way, even commercially, with proper attribution. For these reasons, we cannot publish previously copyrighted maps or satellite images created using proprietary data, such as Google software (Google Maps, Street View, and Earth). For more information, see our copyright guidelines: http://journals.plos.org/plosone/s/licenses-and-copyright.

We require you to either (1) present written permission from the copyright holder to publish these figures specifically under the CC BY 4.0 license, or (2) remove the figures from your submission:

2.1. You may seek permission from the original copyright holder of Figure 2 to publish the content specifically under the CC BY 4.0 license.

We recommend that you contact the original copyright holder with the Content Permission Form (http://journals.plos.org/plosone/s/file?id=7c09/content-permission-form.pdf) and the following text:

“I request permission for the open-access journal PLOS ONE to publish XXX under the Creative Commons Attribution License (CCAL) CC BY 4.0 (http://creativecommons.org/licenses/by/4.0/). Please be aware that this license allows unrestricted use and distribution, even commercially, by third parties. Please reply and provide explicit written permission to publish XXX under a CC BY license and complete the attached form.”

Please upload the completed Content Permission Form or other proof of granted permissions as an "Other" file with your submission.

In the figure caption of the copyrighted figure, please include the following text: “Reprinted from [ref] under a CC BY license, with permission from [name of publisher], original copyright [original copyright year].”

2.2. If you are unable to obtain permission from the original copyright holder to publish these figures under the CC BY 4.0 license or if the copyright holder’s requirements are incompatible with the CC BY 4.0 license, please either i) remove the figure or ii) supply a replacement figure that complies with the CC BY 4.0 license. Please check copyright information on all replacement figures and update the figure caption with source information. If applicable, please specify in the figure caption text when a figure is similar but not identical to the original image and is therefore for illustrative purposes only.

The following resources for replacing copyrighted map figures may be helpful:

USGS National Map Viewer (public domain): http://viewer.nationalmap.gov/viewer/

The Gateway to Astronaut Photography of Earth (public domain): http://eol.jsc.nasa.gov/sseop/clickmap/

Maps at the CIA (public domain): https://www.cia.gov/library/publications/the-world-factbook/index.html and https://www.cia.gov/library/publications/cia-maps-publications/index.html

NASA Earth Observatory (public domain): http://earthobservatory.nasa.gov/

Landsat: http://landsat.visibleearth.nasa.gov/

USGS EROS (Earth Resources Observatory and Science (EROS) Center) (public domain): http://eros.usgs.gov/#

Natural Earth (public domain): http://www.naturalearthdata.com/

--- We followed the suggested option 2.2.(ii) and supplied a replacement figure produced on our computers (the figure caption has been updated with the source information).

Reviewers' comments:

5. Review Comments to the Author

Reviewer #1: This paper extends the classical SIR epidemic spread model by introducing the "quarantined" compartment in the context of COVID-19 spread. In addition, an integer programming model is proposed for optimizing the number of drones to deliver tests within a metropolitan area. The models may be helpful to prevent epidemic spread. However, the proposed SIQR model is not used in the drone fleet optimization model, which makes the two sections independently.
The specification of the optimization model is mainly descriptive. Its formulation is proposed. In addition, the performance of the optimization model should be presented clearly to justify its effectiveness.

--- We singled out the integer program formulation into a separate paragraph, ending with the full statement of the model. We now also indicate the computation times that were needed to solve the optimization problems.

In the Introduction section, more works on trend/severity prediction in the field of public health should be referred to, such as:

Jointly modeling area-level crash rates by severity: A Bayesian multivariate random-parameters spatio-temporal Tobit regression. Transportmetrica A: Transport Science, 2019, 15(2): 1867-1884.
Investigating the impacts of real-time weather conditions on freeway crash severity: A Bayesian spatial analysis. International Journal of Environmental Research and Public Health, 2020, 17(8), 2768.
Spatial joint analysis for zonal daytime and nighttime crash frequencies using a Bayesian bivariate conditional autoregressive model. Journal of Transportation Safety and Security, 2020, 12(4): 566-585.

--- Added the suggested references

There should be a section at the end to summarize the conclusions and the limitations of the research and to draw the directions for future research.

--- Added Conclusions section in the end of the paper

Besides, the format of the manuscript (especially the citations and references) should be revised according to the guidelines of PLOS ONE.

-- We put the paper into PLOS latex template and followed PLOS formatting instructions.

Reviewer #2: The topic of this paper is very timely. It is technically sound and useful for both research and practice. The results and discussion are valid and properly supported. My minor suggestion is that the structure of paper should be adjusted from the current “Introduction-Results-Discussion-Methods”into “Introduction-Methods-Results-Discussion”. The format of references should also be adjusted according to the journal instructions.

--- We rearranged the sections as suggested (making minor text adjustments to ensure terms are defined before first use). We put the paper into PLOS latex template and followed PLOS formatting instructions.
